# Supplementary material for: Participation in youth sports influences sarcopenia parameters in older adults
Source: PeerJ. 2023 Nov 10;11:e16432. doi: 10.7717/peerj.16432 (PMC10642365; doi:10.7717/peerj.16432)
Supplement: Supplemental Information 2 [file peerj-11-16432-s002.pdf]

Spoštovani,

pred Vami je vprašalnik, ki se navezuje na sociodemografske podatke, Vaše splošno zdravstveno stanje ter na Vašo telesno aktivnost. Pozorno preberite vprašanja ter nanje iskreno odgovorite.
Vsi pridobljeni podatki iz vprašalnika bodo anonimni in uporabljeni zgolj v raziskovalne namene.

SOCIODEMOGRAFSKI PODATKI

Obkrožite številko pred ustreznim odgovorom oz. dopolnite manjkajoče podatke:

1. **Katero stopnjo javno veljavne izobrazbe ste dosegli, redno ali izredno?** (*obkrožite*)

1	Brez šolske izobrazbe oz. nepopolna osnovna izobrazba, 1 – 3 razredi
2	Nepopolna osnovna izobrazba, 4 – 7 razredov osnovne šole
3	Osnovna izobrazba (končana osnovna šola)
4	Dvo- ali triletna poklicna izobrazba (npr. obdelovalec kovin, frizer..)
5	Srednja strokovna izobrazba (npr. ekonomski tehnik)
6	Srednja splošna izobrazba (gimnazija)
7	Višja strokovna izobrazba (višješolska izobrazba)
8	Visoka strokovna šola
9	Visoka univerzitetna izobrazba (fakulteta)
10	Specialistična visokošolska izobrazba, magisterij
11	Doktorat

2. **S kom trenutno živite? (***Možnih je več odgovorov, obkrožite vse, ki ustrezajo).*

a	živim sam/sama
b	s partnerjem/partnerko
c	z lastnimi otroki/partnerjevi otroki
D	z vnuki
E	z drugimi sorodniki
f	v domu za starejše, drugi instituciji
G	z nekom drugim? Prosimo, napišite s kom ________________________
H	Ne vem

3. Navedite, kašen je vaš sedanji delovni status (*obkrožite*):

1	Zaposlen (vključeno tudi neplačano delo v družinskem podjetju, status kmeta, gospodinje ipd.)
2	Zaposlen za krajši delovni čas
3	Brezposelna oseba
4	Upokojenec
5	Upokojenec z občasnim delom
3_6	Drugo (navedite):
9	Ne vem

4. Vaše delo (poklic) je potekalo:

1	večinoma sede
2	večinoma stoje
3	kombinirano stoje in sede
9	Ne vem

5. Koliko ljudi vam je tako blizu, da lahko nanje računate, če imate resno osebno težavo?

1	nobeden
2	1 ali 2
3	od 3 do 5
4	več kot 5
9	ne vem

SPLOŠNO ZDRAVSTVENO STANJE

Zdaj pa bi vas radi povprašali o vašem zdravju oz. splošnem počutju.

6. Kako bi ocenili svoje …? *(obkroži številko v vsaki vrsti)*

	zelo dobro	dobro	srednje	slabo	zelo slabo	ne vem
a)Splošno zdravstveno stanje	5	4	3	2	1	9
b)Telesno zmogljivost	5	4	3	2	1	9
c) Psihično počutje	5	4	3	2	1	9
d) Splošno kakovost svojega življenja	5	4	3	2	1	9

7. Kako po vašem mnenju skrbite za svoje zdravje?

1	skoraj nič
2	bolj malo, premalo
3	kar dobro
4	zelo dobro
9	ne vem

8. Ali imate kakšno dolgotrajno bolezen ali dolgotrajno zdravstveno težavo?
(*za dolgotrajno bolezen ali dolgotrajno zdravstveno težavo se šteje taka bolezen/težava, ki traja ali se pričakuje, da bo trajala 6 mesecev ali več.)*

1	da
2	ne
9	ne vem

Pred vami je seznam bolezenskih stanj – preberite podrobno in izpolnite ustrezen odgovor za vsako navedeno bolezen (*izpolnite vsako vrstico*):

		**9.** Ali imate ali ste že imeli katero izmed naslednjih bolezni ali bol. stanj?		**10.** Če DA, ali je diagnozo te bolezni ugotovil zdravnik		**11.** Ali ste imeli to bolezen v zadnjih 12 mesecih in ste zato obiskali zdravnika	
	**Bolezen/ bolezensko stanje**	**DA**	**NE**	**DA**	**NE**	**DA**	**NE**
A	Astma (tudi alergijska astma)	1	2	1	2	1	2
B	Kronični bronhitis, kronična obstruktivna pljučna bolezen, emfizem	1	2	1	2	1	2
C	Srčni infarkt (miokardalni infarkt)	1	2	1	2	1	2
D	Koronarna srčna bolezen (angina pektoris)	1	2	1	2	1	2
E	Zvišan krvni tlak (hipertenzija)	1	2	1	2	1	2
F	Možganska kap (možganska krvavitev, možganska tromboza)	1	2	1	2	1	2
G	Revmatoidni artritis (vnetje sklepov)	1	2	1	2	1	2
H	Osteoartritis (artroza, degenerative bolezni sklepov)	1	2	1	2	1	2
I	Bolečina v križu ali druga kronična okvara hrbta	1	2	1	2	1	2

		Ali imate ali ste že imeli katero izmed naslednjih bolezni ali bol. stanj?		Če DA, ali je <u>diagnozo</u> te bolezni ugotovil zdravnik		Ali ste imeli to bolezen v zadnjih <u>12 mesecih in ste zato obiskali zdravnika</u>	
	**Bolezen/ bolezensko stanje**	**DA**	**NE**	**DA**	**NE**	**DA**	**NE**
J	Bolečina v vratu ali druga kronična okvara vratu	1	2	1	2	1	2
K	Sladkorna bolezen	1	2	1	2	1	2
L	Alergija (npr. rinitis, očesno vnetje, dermatitis, alergija na hrano ali drugo)	1	2	1	2	1	2
M	Razjeda želodca ali dvanajsternika (ulkus)	1	2	1	2	1	2
N	Jetrna ciroza, motnje delovanja jeter	1	2	1	2	1	2
O	Rak (maligni tumor,levkemija,limfom)	1	2	1	2	1	2
P	Močen glavobol, migrena	1	2	1	2	1	2
R	Motnje zadrževanja urina, težave z delovanjem sečnega mehurja	1	2	1	2	1	2
S	Kronična tesnobnost	1	2	1	2	1	2
T	Kronična depresija	1	2	1	2	1	2
J	Druge duševne težave	1	2	1	2	1	2
V	Trajna poškodba ali okvara zaradi posledic nezgode	1	2	1	2	1	2
Z	Druga dolgotrajna bolezen, navedite katera: ___________________________	1	2	1	2	1	2

12.Koliko različnih zdravil trenutno uporabljate? (*navedite število*): ________

13.Navedite katera zdravila jemljete (*navedite vsa, ki jih dobite na recept kot tudi zdravila, ki jih dobite v prosti prodaji*)**:**

____

____

____

____

14. Ali lahko prehodite 2 km (2000 m) po ravnem brez predaha, uporabe palice ali drugega pripomočka za hojo ali pomoči? (*obkrožite številko pred ustreznim odgovorom*)

1	Da, brez težav
2	Da, z manjšimi težavami
3	Da, z večjimi težavami
4	Sploh ne
9	Ne vem

15. Ali lahko hodite po stopnicah navzdol in navzgor brez predaha, uporabe palice ali drugega pripomočka za hojo ali pomoči oz. uporabe stopniščne ograje ? (*obkrožite ustrezno številko*)

1	Da, brez težav
2	Da, z manjšimi težavami
3	Da, z večjimi težavami
4	Sploh ne, ne morem
9	Ne vem

16.Ali se lahko sklonite in pokleknete brez pripomočkov ali pomoči?

1	Da, brez težav
2	Da, z manjšimi težavami
3	Da, z večjimi težavami
4	Sploh ne, ne morem
9	Ne vem

17.Kolikokrat ste v zadnjem letu padli? *(padec je opredeljen kot dogodek, ko se oseba nenadoma in neprostovoljno znajde na tleh ali na drugi, nižji površini)*

1	nikoli
2	enkrat
3	2-3 krat
4	4-5- krat
5	5 in večkrat
9	Ne vem

18.Kdaj ste nazadnje padli? (*obkrožite številko pred odgovorom, ki najbolj natančno opredeljuje čas padca*)

1	še nisem padel
2	v zadnjem letu
3	v zadnjem pol leta
4	V zadnjih treh mesecih
5	v zadnjem mesecu
6	v zadnjem tednu
9	Ne vem

19. Kakšen je bil vzrok padca?

1	še nisem padel
2	vrtoglavica
3	nemoč v nogah (mišična šibkost)
4	spolzka tla
5	neprimerna obleka, obutev
4	stopnice
19.7	drugo:____
9	Ne vem

20.Kako najpogosteje preživljate vaš prosti čas?

	PROSTOČASNA AKTIVNOST	ved no	pogost o	občasn o	redk o	nikol i	ne vem
A	v prostem času se ukvarjam z aktivnostmi, za katere ni potrebna telesna aktivnost (npr. berem, gledam televizijo, grem v gledališče, na obisk k prijateljem ipd.)	5	4	3	2	1	9
B	v prostem času se ukvarjam z lažjimi telesnimi aktivnostmi (npr. se sprehajam, ukvarjam z lažjim delom na vrtu, z lovom, ribolovom, ipd.)	5	4	3	2	1	9
C	v prostem času se ukvarjam z aktivnostmi za ohranjanje telesne kondicije (npr. telovadim, tečem, plavam, igram igre z žogo, smučam ipd.)	5	4	3	2	1	9
D	nikoli nimam prostega časa, ker: ____	5	4	3	2	1	9
E	drugo: ____	5	4	3	2	1	9

GIBALNE NAVADE

Nadalje vas bomo podrobneje spraševali o vaši **telesni aktivnosti.**

Zanima nas telesna dejavnost (gibalna/športna aktivnost), ki je del vašega vsakdana. Spraševali vas bomo o času, ki ste ga preživeli telesno dejavni v tipičnem dnevu ali tednu. Prosimo vas, če lahko odgovorite na vsako vprašanje, tudi če sami sebe ne vidite kot telesno dejavne. Razmislite o aktivnostih, ki so **del vaših opravil doma, na vrtu, del vaše rekreacije oz. športne vadbe** (npr. nordijska hoja, kolesarjenje, balinanje). **Vprašanja se nanašajo na izvajanje tovrstne aktivnosti, na število dni v tednu, ko jo izvajate in na trajanje v dnevu.**

Visoko intenzivna telesna dejavnost se nanaša na aktivnosti, ki zahtevajo **večji telesni napor,** ob katerem se oznojite in ob katerem se pospeši bitje srca in ob katerem dihate precej težje kot normalno (npr. da ste dvigovali težka bremena, kopali v vrtu, hitro tekli, intenzivno kolesarili). Pomislite samo na tako telesno dejavnost, ki ste jo izvajali vsaj **10 minut neprekinjeno.**

Zmerno intenzivna telesna dejavnost se nanaša na aktivnosti, ki zahtevajo **zmerni napor**, ob katerem občutite po telesu toploto in dihate nekoliko težje kot normalno (npr. da ste prenašali lažja bremena, kolesarili v zmernem tempu, tekli v zmernem tempu, zmerno telovadili itd…). V to dejavnost ne vključujte hoje. Pomislite samo na tako telesno dejavnost, ki ste jo izvajali **vsaj 10 minut neprekinjeno.**

Telesna aktivnost pri delu oz. vsakdanjih opravilih

**Aktivnosti pri vsakdanjem delu in opravilih**		
**21.**	Ali so vaše delo oz. opravila zahtevala **visoko intenzivno telesno dejavnost** (npr. da ste dvigovali težka bremena, kopanje ali gradnja itd.), vsaj 10 minut neprekinjeno?	1 DA<br>2 NE
**22.**	**Koliko dni v tipičnem tednu** ste izvajali **visoko intenzivno** telesno dejavnost kot del svojega dela?	Št. dni :______na teden
**23.**	**Koliko časa** ste izvajali **visoko intenzivno** telesno dejavnost v **tipičnem dnevu?**	_______ur, ________min
**24.**	Navedite najdlje trajajočo oz. najpogostejšo **visoko intenzivno telesno dejavnost,** ki ste jo v tipičnem tednu izvajali (npr. sekanje drv…).	

**25.**	Ali je vaše delo zahtevalo **zmerno intenzivno** telesno dejavnost (npr. da ste prenašali lažja bremena,lažja opravila na vrtu, v stanovanju) vsaj 10 minut neprekinjeno. V to dejavnost **ne vključujte hoje?**	1 DA<br>2 NE
**26.**	**Koliko dni** v tipičnem tednu ste izvajali **zmerno intenzivno** telesno dejavnost kot del svojega dela?	Št. dni :_________v tednu
**27.**	**Koliko časa** ste izvajali **zmerno intenzivno** telesno dejavnost v tipičnem dnevu?	_______ur, ________min
**28.**	Navedite najdlje trajajočo oz. najpogostejšo zmerno intenzivno telesno dejavnost, ki ste jo v tipičnem tednu izvajali (npr. vrtnarjenje).	

Aktivni transport

V tem sklopu vas bomo spraševali po telesnih aktivnostih, ki vključujejo **načine transporta**, ki jih koristite v vsakdanjem življenju (pot do trgovine, tržnice, pošte). Aktivnosti, ki ste jo **navedli v predhodnem sklopu ne navajajte ponovno.**

**Gibanje kot sredstvo transporta**			
**29.**	Ali greste peš ali s kolesom vsaj 10 minut neprekinjeno, da pridete doželenega kraja (trgovine, pošte, banke, cerkve)?	1 DA<br>2 NE	
**30.**	**Koliko dni** v tipičnem tednu ste **hodili ali kolesarili** za namenom prevoza vsaj 10 minut neprekinjeno?	Št. dni :________v tednu	
**31.**_	**Koliko časa** ste namenili **hoji ali kolesarjenju** z namenom transporta v tipičnem dnevu?	_______ur, ________min	
**32.**	Navedite katero vrsto aktivnega transporta ste uporabili in kam (npr. v trgovino) ste najpogosteje odšli v običajnem tednu (npr. hoja..).		

Rekreacijske aktivnosti

V naslednjem sklopu **izvzemite delovne aktivnosti in aktivni transport** in se osredotočite na **aktivnosti, ki jih izvajate z namenom rekreacije (gibalne/športne aktivnosti) v prostem času** (sem sodijo tudi organizirana vadba kot tudi individualne telesne aktivnosti)

**Rekreacijske dejavnosti (Gibalno –športne aktivnosti)**		
**33.**	Ali ste izvajali kakšno obliko **visoko intenzivne telesne dejavnosti** (šport, vadba, rekreacijska aktivnost (npr. hiter tek, nogomet, tenis, fitnes)), ki povzroči pospešeno bitje srca in ob katerem dihate precej težje, vsaj 10 minut neprekinjeno.	1 DA<br>2 NE
**34.**	Koliko dni v tipičnem tednu ste izvajali **visoko intenzivno** telesno dejavnost kot del svoje rekreacije (gibalno/ športne aktivnosti, npr. hiter tek)?	Št. dni :_________v tednu
**35.**	Koliko časa ste izvajali visoko intenzivno telesno dejavnost v tipičnem dnevu?	_______ur, ________
**36.**	Navedite najdlje trajajočo oz. najpogostejšo visoko intenzivno telesno dejavnost, ki ste jo z namenom rekreativne gibalne/športne aktivnosti izvajali v tipičnem tednu (npr. tenis..)	
**Rekreacijske dejavnosti (Gibalno –športne aktivnosti)**		
**37.**	Ali ste v tipičnem tednu izvajali kakšno obliko **zmerno intenzivne telesne dejavnosti** (šport, vadba, rekreacijska aktivnost, ki povzroči rahlo povišan srčni utrip in dihanje (npr. hitra hoja, kolesarjenje, plavanje, odbojka) vsaj 10 minut neprekinjeno?	1 DA<br>2 NE (nadaljujte s sklopom C5, vprašanje 16)
**38.**	Koliko dni v tipičnem tednu ste izvajali **zmerno intenzivno** telesno dejavnost kot rekreativno oz. gibalno/športno dejavnost?	Št. dni :________ v tednu
**39.**	Koliko časa ste izvajali **zmerno intenzivno** telesno dejavnost v tipičnem dnevu?	_______ur, ________min
**40.**	Navedite najdlje trajajočo oz. najpogostejšo **zmerno intenzivno telesno** dejavnost, ki ste jo z namenom rekreativne gibalne/športne aktivnosti izvajali v tipičnem tednu (npr.hitra hoja).	

Naslednje vprašanje je namenjeno **navadam sedenja**, ob počitku po delu, doma, ob druženju s prijatelji (vključujoč sedenje za mizo, s prijatelji, gledanju televizije, opravljanje ročnih del, prevozu v avtu, v avtobusu) vendar ne vključuje časa vašega spanja:

SEDENTARNE NAVADE		
**41.**	Koliko časa v **običajnem dnevu sedite** (vključujoč vse naštete oblike sedenja) ?	_______ur, ________min
	**Koliko največ časa v običajnem dnevu presedite skupaj, brez aktivnih odmorov?**	_______ur, ________min

42. V **kakšnih oblikah ste gibalno/športno aktivni?** *(obkrožite številko v vsaki vrsti)*

		vedno	pogosto	občasno	redko	nikoli
A	doma – vadim sam	5	4	3	2	1
B	z družino	5	4	3	2	1
C	S partnerjem	5	4	3	2	1
D	s prijatelji	5	4	3	2	1
E	organizirano v društvu	5	4	3	2	1
F	drugo (navedite): _____________	5	4	3	2	1

43.Telesno aktiven sem (oz. bi bil) zaradi? *(Možnih je več odgovorov, vendar obkrožite največ 3 – tiste, ki imajo za vas največji večji pomen)!*

A	boljše postave
B	zabave, razvedrila
C	sprostitve
D	splošnega zdravja in boljšega počutja
E	preventive in zdravljenja bolezni
F	druženja s prijatelji
G	boljše telesne kondicije
H	Ne vem
I	drugo (navedite):_________________________

Naslednja vprašanja se bodo nanašala na vašo gibalno aktivnost v preteklosti:

44. Ali ste se **v preteklosti rekreativno** ukvarjali s kakšnim športom (npr. pohodništvo, kolesarjenje, smučanje, plavanje)

1	Ne
2	da, bolj redko sem (navedite šport; npr. planinaril):_______________
3	da, občasno (sezonsko, ob vikendih) sem: _____________________
4	da, pogosto (na tedenski ravni) sem: _________________________
5	da, še danes (npr. smučam, kolesarim..)_______________________

45.Od navedenih športov sem opustil (navedite šport) in **razlog zakaj** ga ne izvajate več (npr. zaradi poškodbe, strahu pred padcem…).

**A1**(šport) __________________, prenehal/-a zaradi **A2**____________________
**B1**(šport) __________________, prenehal/-a zaradi: **B2**___________________
**C1** (šport) __________________, prenehal /-a zaradi: **C3**___________________

46.Ali ste se kot otrok in mladostnik aktivno ukvarjali s športom, ste bili član športnega društva oz. kluba, ste obiskovali redne treninge)? Obkrožite najustreznejši odgovor.

1	Ne
2	da, v času osnovne šole
3	da, v času OŠ in srednje šole
4	da v času OŠ, srednje šole in fakultete (oz nekje do 30 leta) …
5	da, dokler se nisem zaposlil/a
6	da, dokler si nisem ustvaril/a družine in dobil/a otrok
7	da, dokler se nisem poškodoval/a
46_8	drugo (navedite) _________________________
9	neprekinjeno sem aktiven vse do danes…

Če ste obkrožili številko 1 pred odgovorom NE, na 47 vprašanje ne odgovarjajte.

47. V katerem športu (navedite največ 2) ste najdlje vztrajali)?

**A1** šport 1: ____ , A2________ let<br>B1 šport 2: ____B2 ________ let
**Kakšni so bili vaši največji dosežki v tekmovalnem športu?**<br>____<br>____<br>____<br>____

48. **Kdo vas je navdušil za šport?**

1	Oče
2	Mati
3	brat/sestra
4	sorodnik (navedite):
5	Prijatelji /sošolci ___
6	drugo (navedite):
9	Ne vem

49. **Kdo vam je ob tem nudil vso potrebno podporo (oprema, navijanje, prevoz)**?

____

____

____

____

50. **Kaj menite, kako je ukvarjanje s športom vplivalo na vas?**

____

____

____

Prišli ste do konca!

Hvala za sodelovanje!

Prosimo, da se držite navodil in izpolnjen vprašalnik pošljete v priloženi kuverti ALI na naslov

ZRS Koper

Garibaldijeva 1

6000 KOPER

Pripis: POST PANGeA
