# Supplementary material for: Participation in youth sports influences sarcopenia parameters in older adults
Source: PeerJ. 2023 Nov 10;11:e16432. doi: 10.7717/peerj.16432 (PMC10642365; doi:10.7717/peerj.16432)
Supplement: Supplemental Information 3 [file peerj-11-16432-s003.doc]

Dear Sir or Madam,

Here is a questionnaire about your socio-demographic data, your general health and your physical activity. Please read the questions carefully and answer them honestly.

All the data obtained from the questionnaire will be anonymous and used for research purposes only.

SOCIO-DEMOGRAPHIC DATA

Circle the number in front of the relevant answer or fill in the missing information**:**

1. **Which level of publicly valid education have you attained? (*circle*)**

| 1 | No school education or incomplete primary education, grades 1-3 |
| --- | --- |
| 2 | No school education or incomplete primary education, grades 4-7 |
| 3 | Primary education |
| 4 | Two or three years' training (e.g. metalworking, hairdressing...) |
| 5 | Secondary vocational education (e.g. economic technician) |
| 6 | Secondary general education |
| 7 | Higher professional qualification (post-secondary education) |
| 8 | Technical college |
| 9 | University degree (faculty) |
| 10 | Specialist higher education qualification, master’s degree |
| 11 | PhD |

1. ***Who are you currently living with?*** *(There are several possible answers, please circle all that apply).*

| a | I live alone |
| --- | --- |
| b | with my partner |
| c | with my own children/partner's children |
| D | with my grandchildren |
| E | with other relatives |
| f | in a retirement home, other institution |
| G | with someone else? Please write with whom ______________________ |
| H | I don't know |

1. **Please state your current employment status (*circle*):**

| 1 | Employed (including unpaid work in family business, status as farmer, housewife, etc.) |
| --- | --- |
| 2 | Part-time employee |
| 3 | Unemployed |
| 4 | Retired |
| 5 | Pensioner with occasional work |
| 3_6 | Other (please specify): |
| 9 | I don't know |

1. **Your work (profession)**:

| 1 | mostly seated |
| --- | --- |
| 2 | Mostly standing |
| 3 | combined standing and sitting |
| 9 | I don't know |

1. **How many people are close enough to you that you can count on them if you have a serious personal problem?**

| 1 | none |
| --- | --- |
| 2 | 1 or 2 |
| 3 | 3 to 5 |
| 4 | more than 5 |
| 9 | don't know |

GENERAL STATE OF HEALTH

Now we would like to ask you about your health or general well-being.

1. **How would you rate your …? (*circle*)**

|  | Very good | Good | Medium | Poor | Very poor | Don't know |
| --- | --- | --- | --- | --- | --- | --- |
| a) General health | 5 | 4 | 3 | 2 | 1 | 9 |
| b) Physical performance | 5 | 4 | 3 | 2 | 1 | 9 |
| c) Mental well-being | 5 | 4 | 3 | 2 | 1 | 9 |
| d) General quality of life | 5 | 4 | 3 | 2 | 1 | 9 |

1. **How do you take care of your health?**

| 1 | almost nothing |
| --- | --- |
| 2 | more little, too little |
| 3 | quite good |
| 4 | very good |
| 9 | I don't know |

1. **Do you have a long-term illness or health problem?**

(A long-term illness or long-term health problem is one that lasts or is expected to last for 6 months or more.)

| 1 | Yes |
| --- | --- |
| 2 | No |
| 9 | Don't know |

**Here is a list of conditions - please read in detail and fill in the appropriate answer for each condition listed (*complete each line*)**:

|  |  | Do you have or have you had any of the following diseases or conditions? | | If YES,  whether the diagnosis was made by a doctor | | Have you had this condition in the last 12 months and seen a doctor for it? | | |
| --- | --- | --- | --- | --- | --- | --- | --- | --- |
|  | **Disease/condition** | **YES** | **NO** | **YES** | **NO** | **YES** | **NO** | |
| A | Asthma (including allergic asthma) | 1 | 2 | 1 | 2 | 1 | 2 | |
| B | Chronic bronchitis, chronic obstructive pulmonary disease, emphysema | 1 | 2 | 1 | 2 | 1 | 2 | |
| C | Heart attack (myocardial infarction) | 1 | 2 | 1 | 2 | 1 | 2 | |
| D | Coronary heart disease (angina pectoris) | 1 | 2 | 1 | 2 | 1 | 2 | |
| E | High blood pressure (hypertension) | 1 | 2 | 1 | 2 | 1 | 2 | |
| F | Stroke (brain hemorrhage, cerebral thrombosis) | 1 | 2 | 1 | 2 | 1 | 2 | |
| G | Rheumatoid arthritis (inflammation of the joints) | 1 | 2 | 1 | 2 | 1 | 2 | |
| H | Osteoarthritis (arthrosis, degenerative joint disease) | 1 | 2 | 1 | 2 | 1 | 2 | |
| I | Low back pain or other chronic back disorder | 1 | 2 | 1 | 2 | 1 | 2 | |
| J | Neck pain or other chronic neck disorder | 1 | 2 | 1 | 2 | 1 | 2 |  |
| K | Diabetes mellitus | 1 | 2 | 1 | 2 | 1 | 2 |  |
| L | Allergy (e.g., rhinitis, eye inflammation, dermatitis, food allergy or other) | 1 | 2 | 1 | 2 | 1 | 2 |  |
| M | Gastric or duodenal ulcer (ulcer) | 1 | 2 | 1 | 2 | 1 | 2 |  |
| N | Cirrhosis of the liver, liver dysfunction | 1 | 2 | 1 | 2 | 1 | 2 |  |
| O | Cancer (malignant tumor, leukemia, lymphoma) | 1 | 2 | 1 | 2 | 1 | 2 |  |
| P | Severe headache, migraine | 1 | 2 | 1 | 2 | 1 | 2 |  |
| R | Urinary retention disorders, bladder problems | 1 | 2 | 1 | 2 | 1 | 2 |  |
| S | Chronic anxiety | 1 | 2 | 1 | 2 | 1 | 2 |  |
| T | Chronic depression | 1 | 2 | 1 | 2 | 1 | 2 |  |
| J | Other mental health problems | 1 | 2 | 1 | 2 | 1 | 2 |  |
| V | Permanent injury or impairment as a result of an accident | 1 | 2 | 1 | 2 | 1 | 2 |  |
| Z | Other long-term illness, please specify: | 1 | 2 | 1 | 2 | 1 | 2 |  |

1. **How many different medicines are you currently taking**? (*state a number*): _______

**Indicate which medicines you take** *(list all prescription medicines as well as over-the-counter medicines)***:**

___________________________________________________________________________

______________________________________________________________________________________________________________________________________________________

1. **Can you walk 2 km (2000 m) on flat ground without taking a break, using a walking stick or other walking aid, or getting assistance?** (Circle the number in front of the appropriate answer)

| 1 | Yes, without a problem |
| --- | --- |
| 2 | Yes, with minor problems |
| 3 | Yes, with major problems |
| 4 | Not at all |
| 9 | I don't know |

1. **Can you walk up and down stairs without taking a break, using a cane or other walking aid or assistance, or using a stair handrail? *(Circle the appropriate number)***

| 1 | Yes, without a problem |
| --- | --- |
| 2 | Yes, with minor problems |
| 3 | Yes, with major problems |
| 4 | Not at all |
| 9 | I don't know |

1. **Can you bend and kneel without aids or assistance?**

| 1 | Yes, without a problem |
| --- | --- |
| 2 | Yes, with minor problems |
| 3 | Yes, with major problems |
| 4 | Not at all |
| 9 | I don't know |

1. ***How many times have you fallen in the last year*** *(a fall is defined as an event where a person suddenly and involuntarily finds himself or herself on the ground or on another lower surface)****?***

| 1 | **Never** |
| --- | --- |
| 2 | once |
| 3 | 2-3 times |
| 4 | 4-5- times |
| 5 | 5 times or more |
| 9 | I don't know |

1. ***When was the last time you fell?*** *(Circle the number in front of the answer that most accurately identifies the time of the fall)*

| 1 | I haven't fallen yet |
| --- | --- |
| 2 | in the last year |
| 3 | in the last six months |
| 4 | In the last three months |
| 5 | in the last month |
| 6 | in the last week |
| 9 | I don't know |

1. **What was the cause of the fall**?

| 1 | I haven't fallen yet |
| --- | --- |
| 2 | vertigo |
| 3 | weakness in the legs (muscle weakness) |
| 4 | slippery ground |
| 5 | inappropriate clothing, footwear |
| 4 | stairs |
| 19.7 | other:______________________________________ |
| 9 | I don't know |

1. **How do you most often spend your leisure activity?**

|  | LEISURE ACTIVITIES | always | often | occasionally | rarely | never | don't know |
| --- | --- | --- | --- | --- | --- | --- | --- |
| A | in my spare time, I engage in activities that do not require physical activity.  (e.g., reading, watching TV, going to the theatre,  visit friends, etc.) | 5 | 4 | 3 | 2 | 1 | 9 |
| B | in my spare time, I engage in light physical activities.  (e.g., walking, light gardening,  hunting, fishing, etc.) | 5 | 4 | 3 | 2 | 1 | 9 |
| C | in my spare time, I do activities to keep fit.  (e.g., exercising, running, swimming,  playing ball games, skiing, etc.) | 5 | 4 | 3 | 2 | 1 | 9 |
| D | I never have free time because:  __________________________________________ | 5 | 4 | 3 | 2 | 1 | 9 |
| E | other: __________________________________________ | 5 | 4 | 3 | 2 | 1 | 9 |

PHYSICAL ACTIVITIES

We will also ask you more detailed questions about your physical activity.

I am going to ask you about the time you spend doing different types of physical activity in a typical week. Please answer these questions even if you do not consider yourself to be a physically active person. Think first about the time you spend doing work. Think of work as the things that you have to do such as paid or unpaid work, study/training, household chores, harvesting food/crops, fishing or hunting for food, seeking employment. [Insert other examples if needed]. In answering the following questions 'vigorous-intensity activities' are activities that require hard physical effort and cause large increases in breathing or heart rate, 'moderate-intensity activities' are activities that require moderate physical effort and cause small increases in breathing or heart rate.

**Physical activity at work or in everyday activities:**

|  | Does your work involve vigorous-intensity activity that causes large increases in breathing or heart rate like [carrying or lifting heavy loads, digging or construction work] for at least 10 minutes continuously? | 1 YES |
| --- | --- | --- |
|  |  | 2 NO |
|  | In a typical week, on how many days do you do vigorous intensity activities as part of your work? | Number of days/week:______ |
|  | How much time do you spend doing vigorous intensity activities at work on a typical day? | _______h, ________min |
|  | Please indicate the longest lasting or most frequent high-intensity physical activity you did in a typical week (e.g., chopping firewood, etc.). |  |
|  | Does your work involve moderate-intensity activity, that causes small increases in breathing or heart rate such as brisk walking [or carrying light loads] for at least 10 minutes continuously? | 1 YES |
|  |  | 2 NO |
|  | In a typical week, on how many days do you do moderate intensity activities as part of your work? | Number of days/week :______ |
|  | How much time do you spend doing moderate intensity activities at work on a typical day? | _______h, ________min |
|  | Please indicate the longest or most frequent moderate-intensity physical activity you did in a typical week (e.g. gardening). |  |

**Travel to and from places**

The next questions exclude the physical activities at work that you have already mentioned. Now I would like to ask you about the usual way you travel to and from places. For example to work, for shopping, to market, to place of worship. Do not repeat an activity that you mentioned in the previous section.

|  | | | |
| --- | --- | --- | --- |
|  | Do you walk or use a bicycle (pedal cycle) for at least 10 minutes continuously to get to and from places? | 1 YES |  |
|  |  | 2 NO |  |
|  | In a typical week, on how many days do you walk or bicycle for  at least 10 minutes continuously to get to and from places? | Number of days/week:______ |  |
|  | How much time do you spend walking or bicycling for travel on a typical day? | _______h, ________min |  |
|  | Please indicate which type of active transport you used and where (e.g., to the shops) you went most often in a typical week (e.g. walking...). |  |  |

**Recreational activities**

**The next questions exclude the work and transport activities that you have already mentioned. Now I would like to ask you about sports, fitness and recreational activities (leisure),**

|  | Do you do any vigorous-intensity sports, fitness or recreational (leisure) activities that cause large increases in breathing or heart rate like [running or football] for at least 10 minutes continuously? | 1 YES |
| --- | --- | --- |
|  |  | 2 NO |
|  | In a typical week, on how many days do you do vigorous intensity sports, fitness or recreational (leisure) activities? | Number of days/week:______ |
|  | How much time do you spend doing vigorous-intensity sports, fitness or recreational activities on a typical day? | _______h, ________min |
|  | Please indicate the longest lasting or most frequent high-intensity physical activity you did in a typical week for the purpose of recreational physical/sport activity (e.g. tennis...). |  |
|  | Do you do any moderate-intensity sports, fitness or recreational (leisure) activities that cause a small increase in breathing or heart rate such as brisk walking, [cycling, swimming, volleyball] for at least 10 minutes continuously? | 1 YES |
|  |  | 2 NO |
|  | In a typical week, on how many days do you do moderate intensity sports, fitness or recreational (leisure) activities? | Number of days/week :______ |
|  | How much time do you spend doing moderate-intensity sports, fitness or recreational (leisure) activities on a typical day? | _______h, ________min |
|  | Please indicate the longest lasting or most frequent moderate intensity physical activity you did in a typical week (e.g., brisk walk) for the purpose of recreational physical activity/sport. |  |

The following question is about sitting or reclining at work, at home, getting to and from places, or with friends including time spent sitting at a

desk, sitting with friends, traveling in car, bus, train, reading, playing cards or watching television, but do not include time spent sleeping.:

| **SEDENTARY BEHAVIOUR** | | |
| --- | --- | --- |
|  | How much time do you usually spend sitting or reclining on a typical day? | _______h, ________min |
|  | What is the maximum amount of time you sit together in a typical day, without active breaks? | _______h, ________min |

1. ***What forms of physical activity/sports do you participate in?*** *(Circle the number in each row)*

|  |  | always | often | occasionally | rarely | never |
| --- | --- | --- | --- | --- | --- | --- |
| A | at home – by myself | 5 | 4 | 3 | 2 | 1 |
| B | with family | 5 | 4 | 3 | 2 | 1 |
| C | With a partner | 5 | 4 | 3 | 2 | 1 |
| D | with friends | 5 | 4 | 3 | 2 | 1 |
| E | organized in the association | 5 | 4 | 3 | 2 | 1 |
| F | Other (please specify): _____________ | 5 | 4 | 3 | 2 | 1 |

1. ***I am (or would be) physically active because of?*** *(Several answers are possible, but circle no more than 3 - the ones that have the most meaning for you)!*

| A | better figures |
| --- | --- |
| B | fun, entertainment |
| C | relax |
| D | general health and well-being |
| E | prevention and treatment of illness |
| F | socializing with friends |
| G | better physical condition |
| H | I don't know |
| I | other: ________________________ |

**The following questions will ask about your physical activity in the youth**:

1. Have you done any recreational sports in the past (e.g., hiking, cycling, skiing, swimming)?

| 1 | No |
| --- | --- |
| 2 | Yes, more rarely (specify sport; e.g. hiking):______________ |
| 3 | Yes, occasionally: ____________________ |
| 4 | Yes, often (weekly): ________________________ |
| 5 | Yes, still do (e.g. skiing, cycling)______________________ |

1. Of the sports listed, I have given up (please specify the sport) and the reason why you no longer do it (e.g. injury, fear of falling, etc.)

| **A1**(sport) _________________, stopped because of **A2**___________________ |
| --- |
| **B1**(sport) _________________, stopped because of **B2**__________________ |
| **C1** (sport) _________________, stopped because of **C3**__________________ |

1. **Were you active in sport as a child or adolescent, were you a member of a sports association or club, did you regularly participate in training sessions?** Please circle the most appropriate answer.

| 1 | No |
| --- | --- |
| 2 | Yes, during primary school |
| 3 | Yes, during primary and secondary school |
| 4 | Yes, during primary school, secondary school and college (or somewhere up to the age of 30) ... |
| 5 | Yes, until I got a job |
| 6 | Yes, until I started a family and had children |
| 7 | yes, until I got injured |
| 8 | other (please specify) ________________________ |
| 9 | I have been continuously active until today... |

**If you circled 1 before NO, please do not answer question 47.**

1. **Which sport did you participate in the longest?**

| **A1** sport 1: ______________________________________, A2________ years  B1 sport 2: ______________________________________B2 ________ years |
| --- |
| **What have been your greatest achievements in competitive sport?** ________________________________________________________________________________________________________________________________________________________________________________________________________________________________________________________________________________ |

1. **Who inspired you to take up sport?**

| 1 | Father |
| --- | --- |
| 2 | Mother |
| 3 | Brother/sister |
| 4 | relative (please specify): |
| 5 | Friends / classmates ___ |
| 6 | Other (specify): |
| 9 | I don't know |

1. **Who gave you all the support you needed (equipment, winding, transport)?**

______________________________________________________________________________________________________________________________________________________

______________________________________________________________________________________________________________________________________________________

1. **How do you think taking part in sport has affected you?**

_________________________________________________________________________________________________________________________________________________________________________________________________________________________________

You have come to the end!

Thank you for your participation!

Please follow the instructions and send the completed questionnaire in the enclosed envelope OR to the following address.

ZRS Koper

Garibaldijeva 1

6000 KOPER

POST PANGeA
